# Evaluating a Psychodynamic Multifamily Group for Severe Mental Disorders: Baseline Assessment and Methodological Design

**DOI:** 10.1192/j.eurpsy.2026.11736

**Published:** 2026-07-31

**Authors:** S. Castelao-Almodovar, E. de Miguel Aldea, M. M. López

**Affiliations:** Centro de Salud Mental El Escorial, El Escorial, Spain

## Abstract

**Introduction:**

Multifamily group interventions are increasingly recognized as valuable complementary approaches for individuals with severe mental disorders. These groups provide a space where patients and their relatives can jointly explore relational dynamics and emotional experiences. However, systematic evaluations of psychodynamic and group-based multifamily interventions using standardized instruments remain scarce.

**Objectives:**

To describe the methodological design of a prospective evaluation of a psychodynamic multifamily therapy group in a public mental health service, and to present the baseline assessment plan prior to longitudinal follow-up.

**Methods:**

The group includes patients with SMD—mainly psychotic and bipolar disorders—and their relatives, who typically present with neurotic structures and symptoms such as anxiety or depression. This heterogeneity allows exploration of contrasting subjective positions within the same therapeutic space. The intervention follows a psychodynamic group framework, integrating multifamily, group-analytic, and operative group perspectives, and is offered as a complement to standard psychiatric and pharmacological treatment. At the beginning of the therapeutic course (September 2025), participants completed: (a) a sociodemographic questionnaire; (b) the WHOQOL-BREF (quality of life); (c) the Reflective Functioning Questionnaire (RFQ-8); and (d) the short version of the Family Relations Scale (ERI). At the end of the course (June 2026), additional measures will include a Satisfaction Survey and Yalom’s Therapeutic Factors Questionnaire.

**Results:**

Data collection is ongoing. At the time of abstract submission, only baseline questionnaires have been distributed and responses are being gathered. Full descriptive results from the initial assessment will be presented at the congress poster session. Comparative pre–post analyses will be conducted after the completion of the therapeutic cycle in June 2026.

**Conclusions:**

This project introduces a structured methodology to evaluate the impact of psychodynamic multifamily groups in routine psychiatric practice. The integration of validated psychometric tools with a group-based intervention highlights the feasibility of assessing both clinical and relational outcomes. Presenting baseline findings and methodological design at this stage contributes to the evidence base for multifamily interventions as meaningful complements to pharmacological treatment in SMD.

**Disclosure of Interest:**

None Declared

